# “*Our parents kiss in front of us*”: Reasons for early sexual debut among in-school youth in the Manzini Region in Eswatini

**DOI:** 10.1371/journal.pone.0282828

**Published:** 2023-03-10

**Authors:** Nomathemba C. Nxumalo, Mduduzi Colani Shongwe, Nontobeko Gwebu, Cebisile Ngcamphalala, Bonisile S. Nsibandze, Rejoice Nkambule, Harriet Nuwagaba-Biribonwoha

**Affiliations:** 1 Health Research Training Program (HRTP), ICAP at Columbia University, Mbabane, Eswatini; 2 Faculty of Health Sciences, Department of Community Health Nursing Science, University of Eswatini, Mbabane, Eswatini; 3 Faculty of Health Sciences, Department of Midwifery Science, University of Eswatini, Mbabane, Eswatini; 4 ICAP at Columbia University, Mailman School of Public Health, Mbabane, Eswatini; 5 Faculty of Health Sciences, Department of General Nursing Science, University of Eswatini, Mbabane, Eswatini; 6 Ministry of Health, Government of the Kingdom of Eswatini, Mbabane, Eswatini; 7 Department of Epidemiology, Mailman School of Public Health, Columbia University, New York, New York, United States of America; University of the Western Cape, SOUTH AFRICA

## Abstract

**Background:**

Early sexual debut (i.e., sex before the age of 15 years), especially if it is unprotected, may increase the risk of acquiring HIV, sexually transmitted infections, and unwanted pregnancies. We investigated the reasons for early sexual debut among in-school youth in Eswatini, a setting with high HIV incidence among youth.

**Methods:**

This was a qualitative, exploratory-descriptive study whereby data were collected from 81 sexually active in-school youth through seven focus group discussions (FGDs) in four purposively selected public high schools (two urban and two rural) in the Manzini region, Eswatini. In each school, except one, two FGDs (one for boys and one for girls) were conducted. Qualitative data were coded and analyzed thematically in Dedoose version 8.2.14.

**Results:**

Nearly 40% of the participants reported having initiated sexual activity before 18 years. Six major themes emerged from the data: i) Intrapersonal factors (feeling mature, religiosity, nutritional or dietary patterns); ii) Parenting and household factors (living arrangement, lack of sexuality education, working parents, negative role-modeling from adults); iii) Peer and partner pressure (pressure from friends, threats from sexual partners, intergenerational sexual partnerships and transactional sex, testing sexual prowess, desire to fit in); iv) Contextual factors (neighborhood, location); v) Mass media (cell phone ownership, social media, and television shows or movies); and vi) Cultural factors (attending traditional ceremonies, loss of cultural norms, values, and traditions, and dress code).

**Conclusion and recommendations:**

The poor monitoring and negative role-modeling by elders highlight the importance of involving parents or guardians as key stakeholders when designing interventions targeting risky sexual behavior among youth. The multifaceted nature of the cited reasons for early sexual debut calls for interventions aimed at curbing risky sexual behavior to be culturally sensitive and responsive to the themes identified in this study.

## Introduction

Adolescence (10–19 years) is a period riddled with challenges of physical and emotional development, of which some of the decisions and actions taken during this period can have lifetime consequences [1]. One such common decision adolescents often face is when to initiate sexual intercourse (i.e., sexual debut). The appropriate age to initiate sexual activity varies by culture and context, but the general expectation is that adolescents may begin sexual activity after attaining the legal age of sexual consent, which, however, also varies from country to country. While the age of sexual consent is not expressly stipulated in most countries in Africa, in East and Southern Africa (ESA), it ranges from 13 years in Comoros to 18 years in many countries [2], including in Eswatini [3]. On the contrary, in Europe, all countries have set legal minimum age limits for initiating sexual relations, and none have it lower than 14 years [4].

There is no agreement on the definition of early sexual debut in literature [5–7], but it is generally defined as initiating sexual intercourse before the age of 15 years [8–10]. In South Africa, the prevalence of early sexual debut is higher amongst males (12%) than females (6%) [11], whereas in Eswatini it stands at 3.5% among young people aged 15–24 years (4.5% among males and 2.5% among females) [12,13], with rural females and urban males said to become sexually active earlier than their urban and rural counterparts, respectively [14,15]. Early sexual debut puts youth at increased risk of unplanned pregnancies and HIV infection [1,16]. For example, in 2013, about 380 000 new HIV infections, globally, were attributed to early sexual intercourse [17] whereas, in Eswatini, 18.7% of males and 50.6% of females aged 15 years and older who had a sexual debut before the age of 15 years were living with HIV [12]. In Eswatini, youth who had an early sexual debut have higher odds of engaging in risky sexual behaviors than their counterparts [18], and 87 in 1000 adolescents aged 15–19 are mothers [13]. Engaging in early sexual debut also puts youth at risk of sexual abuse and delinquent behavior [19], which curtail continued education, especially for adolescent girls. Therefore, delaying sexual debut offers protection from negative sexual and reproductive health outcomes, such as STIs, HIV [20,21], and sexual violence [21,22].

Factors contributing to early sexual debut differ by country, culture, and context, with adolescents in developing countries more likely to experience sexual debut earlier than their counterparts in developed countries [23]. Other risk factors include being less educated adolescents [24], being male, having friends who engaged in sexual activities, alcohol intake [25], living in a polygamous family, peer sex education, poor reproductive health knowledge [9], poor parental monitoring, cigarette smoking, not attending religious programs, and exposure to pornographic materials [26]. However, there is a dearth of qualitative research investigating the reasons for youth engaging in sexual intercourse earlier than the legal age of sexual consent. Utilizing a qualitative methodology helps to unearth an in-depth understanding of phenomena within a society thus providing policy decision-makers with rich data that they can use to inform youth sexual and reproductive health programs [27]. As a result, we conducted this study to explore the reasons for in-school youth to initiate sexual activity earlier than 18 years in Eswatini.

## Materials and methods

### Study design and setting

Eswatini is a small land-locked country in Southern Africa with a population of about 1.1million, 20.7% of whom are youth aged 15–24 years [28]. The country is predominantly Christian (89.3%) [28], a religion that strongly condemns premarital sex. Eswatini has highly conservative cultural norms and values that are highly safeguarded and widely practiced, especially in rural areas, where a majority of the population resides. For example, in Eswatini culture, premarital sex is strongly condemned, while chastity is encouraged and promoted through various traditional practices such as the annual ceremonies *Umhlanga* (Reed) Dance for girls and *Lusekwane* (named after a sacred shrub) for boys where they celebrate their chastity [29]. Despite these traditional and religious practices, the youth still engage in early sexual debut, raising questions on the factors underlying their sexual behaviors [30].

We employed a qualitative research approach, using an exploratory-descriptive study design, and collected data from in-school youth in June-July 2019. The data were collected from four purposively selected high schools (two urban and two rural) within the Manzini region, the central and most populous region in Eswatini. The schools were chosen because they are public schools with high enrolment numbers in the region, they are non-gender-segregating, and are non-faith-based schools.

### Study population, sampling, and sample size

Eligibility for participation in this study was limited to youth aged 18 to 24 years, who were day scholars (non-boarders) from schools in the Manzini region, who were unmarried but had had sexual intercourse. We excluded students younger than 18 years because they would have required parental consent to participate in the study, yet the subject matter (sexual behavior) is considered sensitive and would have resulted in the disclosure of their sexual behaviors to their parents since we targeted only youth who had had sex. In each school, the headteacher facilitated that all Form 4 and 5 (Grade 11 and 12) students gather in one class where we approached them as a group. Once in the room, we first explained to the students (without divulging what the study was about) that we were conducting a study among 18-24-year-old students, and politely asked all other students to be excused. Thereafter, we purposively selected students who reported previous sexual debut by administering a brief screening questionnaire with close-ended questions (S1 Text) asking the participants’ socio-demographic characteristics and whether they have had sex or not. Participants were made to self-count from one up to the last number and were asked to write that number on the top right corner of the first page of the screening questionnaire and to memorize it which would then be their study number.

Once they had filled out the screening questionnaire, the research assistants (RAs) quickly scanned through the questionnaires in a separate room, setting aside all the screening questionnaires of those who recorded that they had had sex, while the students waited in the classroom. After that, the RAs went back to the classroom and read out all the pseudo names (i.e., the numbers they had written on the brief questionnaire) for those who had ever had sex and asked them to remain behind while the rest were excused. The students were not told that they have been asked to remain behind because they had had sex, to avoid disclosure of their sexual behaviors to the other students. At this point, the purpose of the study and all other study information was explained to the students, and thereafter invited to participate. Those who agreed were asked to remain behind where-upon a date was set for the focus group discussions (FGDs) to be conducted. However, in some schools, at the request of the school administration, citing time constraints, the FGDs were conducted on the same day of sampling. The FGDs were homogenous i.e., the participants were divided by gender and interviewed separately. In one school though, where all the girls who had earlier agreed to participate, the same did not show up on the day of the FGD, hence, there was no FGD conducted among girls in that school. As a result, 81 students (42 boys and 39 girls) participated in the seven FGDs in all schools combined, with each FGD having an average of 11 participants.

### Data collection process

A semi-structured interview guide was used to collect data through the FGDs (S2 Text). The guide had one broad question asking participants to give reasons why youth initiate sex earlier than 18 years in Eswatini. Facilitators used a job aid to solicit more information from each participant in the FGDs, while predetermined global probes were used to explore potential reasons under dimensions identified in the literature. Examples of the global probes used include, “*Please explain more on*…*; Would you tell us more about …*.*; What do others think about what was raised by Participant X …; What other reasons can you put forward…”*, *depending on the response of the participants prior to each probe*. During the discussions, participants were never asked to state to the group whether they had had sex or when they started having sex to avoid disclosure. The facilitators posed the broad questions and thereafter allowed the discussions to be driven by the participants among themselves. Unique pseudonyms were used to ensure that during the discussion participants got a fair opportunity to express their views. A voice recorder was used to record all discussions. Before data collection commenced, a one-day training was conducted for the facilitators on qualitative research methods, research ethics, and conducting FGDs, including role-plays. On average, each FGD lasted for about one hour.

### Study rigor

To ensure study rigor, we employed Lincoln and Guba’s [31,32] trustworthiness criteria, such as conducting member checking for findings that were ambiguous to capture the real meaning of the participants’ responses to ensure the credibility of the findings. Not involving teachers in the data collection process and using non-coercion when inviting students to participate in the study also ensured the credibility of the findings [33]. We provide descriptions of the study setting and sample characteristics to enhance the transferability of the study findings. To ensure confirmability, we kept all documentation regarding the step-by-step conduct of the study to allow the conduct of an audit trail [32]. Lastly, to ensure the authenticity of the findings, we retained the use of local language expressions for some of the findings in the participants’ quotes. The study is reported following the Consolidated criteria for Reporting Qualitative research (COREQ) 32 checklist (S3 Text) [34].

### Data analysis

Descriptive statistics on participants’ background characteristics were computed using Statistical Package for Social Sciences (SPSS) version 20 [35], whereas qualitative data were analyzed in Dedoose version 8.2.14 [36], a qualitative data analysis software program. Data for boys were analyzed first as the initial plan was to present the results separately for each gender, however, after both analyses, there were no overt differences in the responses of the two groups, hence a decision was made to present them concurrently. Data analysis began after all FGDs had been completed. Transcribers with prior transcription experience first transcribed the audio files into SiSwati and thereafter translated them into English. Three researchers first read each transcript to familiarize themselves with the texts and to immerse themselves in the data.

During the second reading, the second author carefully examined the texts and categorized them to formulate a coding scheme. Once the initial coding scheme had been developed, the research team met to discuss the proposed codes and reached a consensus, with a few refinements, integration of some of the codes, and categorizing them into broad themes. Thereafter, the English transcripts were uploaded into Dedoose where they were coded independently by the second author by assigning the codes to pieces of texts based on the coding scheme agreed-to and approved by the research team, prior.

### Ethical considerations

The study protocol was approved by the Eswatini Health and Human Research Review Board of the Ministry of Health and the Columbia University Institutional Review Board. Administrative permission to enter schools was granted by the Ministry of Education and Training, and the principals of the different schools. Participants provided written informed consent prior to participation in the FGDs, including consent to be audio-recorded. Participants were assured of their right to withdraw from participation at any time and that any information shared with the research team would not be shared with the school administration or their teachers.

## Results

### Participant characteristics

A majority of the participants were boys (51.9%, *n* = 42/81), were aged between 20–24 years (56.6%, *n* = 43/76), and were from rural areas (79.7%, *n* = 63/79). Nearly 40% (*n* = 31/79) reported having initiated sexual activity before 18 years (**Table 1**).

**Table 1:** Participants’ background characteristics (*N* = 81).

*Characteristic*	*n*	*%*
***Age in years (n = 76)a***		
***18–19***	***33***	***43*.*4***
***20–24***	***43***	***56*.*6***
***Gender***		
***Boys***	***39***	***51*.*9***
***Girls***	***42***	***48*.*1***
***School code***		
***School 1***	***18***	***22*.*2***
***School 2***	***5***	***6*.*2***
***School 3***	***36***	***44*.*4***
***School 4***	***22***	***27*.*2***
***Grade***		
***Form 4 (Grade 11)***	***44***	***54*.*3***
***Form 5 (Grade 12)***	***37***	***45*.*7***
***Residence (n = 79)a***		
***Urban***	***16***	***20*.*3***
***Rural***	***63***	***79*.*7***
***Religion***		
***Christian***	***80***	***98*.*8***
***Other***	***1***	***1*.*2***
***Living arrangement (n = 78)a***		
***Both parents***	***16***	***20*.*5***
***Father only***	***9***	***11*.*5***
***Mother only***	***28***	***35*.*9***
***Guardian***	***20***	***25*.*6***
***Child-headed/alone***	***5***	***6*.*4***
***Family size***		
***≤3 people***	***13***	***16*.*0***
***4–6 people***	***45***	***55*.*6***
***≥ 7 people***	***23***	***28*.*4***
***Average pocket money per month (US$ 1 = SZL15*.*20)***		
***SZL0***	***12***	***14*.*8***
***SZL1-49***	***39***	***48*.*2***
***SZL50+***	***30***	***37*.*0***
***Age at sexual debut (n = 79)a***		
***<18 years***	***31***	***39*.*2***
***≥18 years***	***48***	***60*.*8***
***Nature of first sex***		
***Voluntary***	***80***	***98*.*8***
***Forced***	***1***	***1*.*2***

*Notes*: *SZL*: *Swaziland Lilangeni*, *abbreviated as E;* ^*a*^*The n is less than 81 due to missing data*.

### Reasons for early sexual debut

We grouped the themes that emerged from the data into six categories: i) Intrapersonal factors (feeling mature, religiosity, nutritional or dietary patterns); ii) Parenting and household factors (living arrangement, lack of sexuality education, working parents, negative role-modeling from adults); iii) Peer and partner pressure (pressure from friends, threats from sexual partners, intergenerational sexual partnerships and transactional sex, testing sexual prowess, desire to fit in); iv) Contextual factors (neighborhood, location); v) Mass media (cellphone ownership, social media, and television shows or movies); and vi) Cultural factors (attending traditional ceremonies, loss of cultural norms, values, traditions, and dress code). **Table 2** summarizes the themes and subthemes.

**Table 2:** Reasons for early sexual debut among in-school youth in Eswatini.

Theme	Sub-theme	Frequency^a^
Intrapersonal factors	• Feeling matured/grown-up	4
	• Nutrition/diet	4
	• Low sexual self-efficacy	4
	• Religiosity (attending church activities)	3
Peer and partner pressure	• Desire to fit in	41
	• Sexual prowess	16
	• Fear of being labeled impassive and pressure from girls	13
	• Sex strengthens relationship bond	11
	• Desire to be relevant in conversations about sex	7
	• Threats from sexual partners	6
Family/household factors	• Poverty	41
	• Living arrangement	28
	• Transactional sex (intergenerational sexual partnerships)	22
	• Negative role modeling by parents and older siblings	20
	• Poor parental monitoring e.g., working parents	16
	• Lack of sexuality education	15
	• Pressure from parents and older siblings	8
Contextual factors	• Area of residence, changing living environment/neighborhood	19
	• Attending social gatherings e.g., nightclubs, parties, soccer games	14
	• Substance abuse in the neighborhood	12
Mass media	• Television, cellphone ownership, and social media	37
Cultural factors	• Attending traditional ceremonies	35
	• Girls’ revealing dress code	16
	• Loss of cultural norms and values	5

*Notes*: ^*a*^*Number of times theme or sub-theme was mentioned by respondents in all FGDs combined*.

### Theme 1: Intrapersonal factors

Participants mentioned several reasons why youth engage in early sexual debut, including considering themselves grown-up or feeling mature enough to start having sex; the type of food they eat which they said makes them experience menarche early and start having sexual desires; low sexual self-efficacy; and religiosity (free time during church-related activities):

*When we get to Forms 4 and 5*, *we see ourselves as adults and start dating and having sex*. *We tell ourselves that it’s okay because we are now grown up–*Girl, FGD 4

*We eat a lot of eggs*, *cheese*, *etc*., *which make our bodies react quickly sexually*–Boy, FGD 7

*Some of us cannot control ourselves… we are weak; we don’t have control over our bodies*–Boy, FGD 7

*Our parents always allow us to go to church-related activities such as all-night prayers… Along the way*, *we meet with our friends and our sexual partners whom we would have made arrangements to meet… We will have a chance to engage in sex–*Boy, FGD 7

### Theme 2: Family/Household factors

Participants mentioned that coming from a poor household, either as a result of the death of a parent or breadwinner, or due to their parents being unemployed, forces them to engage in transactional sexual relationships and intergenerational sex partnerships, yet in such partnerships, it is difficult for them to negotiate sex. They also mentioned that coming from a child-headed home and staying alone afford them ample unsupervised time, and that staying in a big house or in one-room flats also put them at risk of having early sex:

*It is the poor family background*, *such as when the parents died or are not working… we start having relationships early so that we can get money*. *However*, *for the person to give me money*, *I need to have sex with him because I have to satisfy his pleasures–*Girl, FGD 4

*We see our friends coming from nightclubs with cash and tell us that “the man just touched me and gave me money*, *I gave it [had sex] to him just this once in the bushes and got E500… every day they will be counting cash*… *We end up trying it too and find ourselves having sex at a young age—Girl*, FGD 3

*Some of the families are child-headed*: *there are no adults to instill family rules… there is no one supervising the children; they do as they please*, *such as coming back late at night–*Boy, FGD 7

*Staying alone pushes us to engage in sex early because there are high chances of bringing a girlfriend at any time as there is no one to reprimand you–*Boy, FGD 6

*You find that we all live in one big house*, *or rented flats*, *or staff houses… there is a bathroom and toilet inside*, *and we cross each other along the passages*. *For example*, *I sometimes see my sister covered only in a towel and my mother coming out of her bedroom wearing her gown only; mind you*, *I am now a grown-up boy; I will start imagining…thinking about what I saw… obviously*, *I will want to go and taste it somewhere–*Boy, FGD 2

In all FGDs, participants mentioned that parents do not monitor what they do during their spare time, especially working parents as they rarely have time with them because they either return late from work every day or are always on their laptops or gadgets’, even when they are in the house:

*Some parents don’t care when the child left home*, *when they returned*, *or how many days they have been away… For example*, *I can report to my parents that I am going to study at my friend’s place*, *and ask to sleep over there… My parent would not even make a follow-up to confirm if I really went there…*. *At times you find that our parents work in towns*, *and we live with our grandparents; they don’t monitor us*, *so we go out and do as we please–*Girl, FGD 4

In all the FGDs, participants blamed parents for not giving them sexuality education. They said that leaves them with no choice but to learn everything from their peers, who, however, never warn them about the dangers of early sex, instead, they encourage them to ‘go taste how it (sex) feels’:

*Parents do not talk to us about sex… some parents feel like once they talk to us about sex it will be like they are “sending us” to go and have sex*, *yet that is not the case; we need to learn and know these things*–Girl, FGD 4

*In many homes*, *parents do not sit down with us to talk about sexuality issues… As a result*, *we end up relying on our friends for such information*, *yet they mislead us–*Boy, FGD 2

Surprisingly, in some FGDs, participants mentioned that they started engaging in sexual activities early because their parents and/or older siblings pressurized them to start romantic relationships earlier than they wished. Boys stated that their fathers ask them if they have started proposing love to girls and if they say no, the fathers would be disappointed. On the other hand, girls mentioned that their mothers would also tell them to look for alternative financing sources to support themselves as they (parents) do not have money to meet all their needs. The youth said that they interpreted that to mean they have to start romantic relationships to get financial support. To prove that their perception is correct, when they bring the groceries home, their parents never ask where they took those groceries from:

*At times the mother will be the one encouraging the daughter to go out… she may even ask her*: *“What are we going to eat if you don’t go out”*? *She will tell her to bath*, *get dressed*, *look beautiful*, *and go to the shops so that boys can see her*. *Sometimes it is our parents that encourage us… because*, *when we return with plastic bags*, *they do not ask any questions since they know that at least they will have something to eat that day–*Girl, FGD 4

*Some of our fathers ask us if we have started proposing love to girls and if you say no*, *you will hear your dad saying*: *“Oh*! *You don’t have any girlfriend up to now*? *It’s a disgrace*, *you are not like me*.*” We interpret it to mean he is ok if I go and do it (sex)–*Boy, FGD6

*Most of the time you find that when you consult your brother for dating advice*, *he will say*: *“take the girl and have sex with her as a sign that she truly loves you*, *and for you to be rest-assured that she is truly yours*.*”–*Boy, FGD 6

Participants also expressed that they engage in early sexual activities because they see their parents or older siblings bringing mistresses and extramarital partners to their houses, and at times kiss in front of them, which makes them also want to experiment what they saw their parents or older siblings doing:

*If they (parents/older siblings) do not practice what they teach me*, *I would follow in their footsteps… I will also go to my boyfriend and come back the following day–*Girl, FGD 5

*If my parents bring multiple partners to the house*, *even if they can sit down with me and advise me against such behaviors*, *I will just say to myself*: *“What is she/he telling me because she is doing the very same thing in front of me*?*” I will be angry at him or her and start overdoing things to try and revenge on him or her so that he/she can feel the pain of seeing me doing what he warned me against… I would want him/her to see how I also feel when he/she brings a lover into the house in our full view–*Girl, FGD 5

*Our parents kiss in front of us…they have sex in the next room*, *and you hear the sounds… They make us want to do it too*. *In the past*, *this was not common as there were separate houses for parents and children–*Boy, FGD 1

### Theme 3: Peer/Partner pressure

In all the FGDs, almost every participant mentioned peer pressure as the number one reason they engaged in early sex. They mentioned several reasons such as that they desire to live luxurious and extravagant lifestyles like their counterparts from rich families. Some said that they do it to stay relevant in conversations about sex with their friends; to strengthen their relationship bonds with their sexual partners as advised by their peers; to experiment or ‘taste what is in there (under the mini-skirt)”; to brag to their peers that they have also had sex; due to fear of being labeled impassive by their girlfriends; and due to pressure from girls in their social cycle. Girls further stated that at times their sexual partners pressurize them to engage in sex early by threatening to kill them if they refuse:

*We want to impress friends*… *They (peers) tell us that sex is nice*, *and mind you*, *you are still a virgin at that time… and when you say*, *“I am still a virgin”*, *my friends will say*, *“you are a fool*, *you don’t know anything*, *go and have sex so that you can experience it”*. *That will push me to go and have sex so that I experience what they feel—*Girl, FGD 3

*I see all my friends wearing nice and expensive clothes*, *yet I don’t have those clothes because I can’t afford their living standard…my friends will tell me how they get these clothes—from their boyfriends… Oblivious*, *I will then do what my friends do*, *yet the boy will want something in return*–Girl, FGD 5

*If he has upset you and you want to leave him (boyfriend)*, *he will just say*: *“I will kill you*! *Why do you want to leave me*?*” And you find that you do not even know his parental home*, *and you do not even know his real name at times*… *He can even tell you to recount all the things he has been buying you all along and tell you to repay all of them*–Girl, FGD 4

*As young people*, *we talk about a lot of topics*, *including sex… As a virgin*, *there is nothing you can comment about under that topic since you won’t be having any experience with that…*. *definitely*, *after such conversations you also want to go and ‘taste’ so that you can comment the next time they are discussing the topic*–Boy, FGD 6

*Sometimes boys tell us that what makes the bond [love] to be strong is sex… If I do not have sex with him*, *he will leave me because our love is not strong; he will go and get it elsewhere*… *Some boys will say*: *“if you don’t have sex with me*, *it means you don’t love me; you need to ‘sleep’ with me so that I can see that you truly love me*.—Girl, FGD 3

*Those who have already done it [sex]*, *do not tell us the truth*, *or rather*, *they do not portray sex in such a way that will make us feel discouraged to engage in it*. *For example*, *they won’t even tell you it was so painful the first time… All they will say is*: *“Yiiii*! *It was really nice; you can’t imagine how sweet it was my friend” … In that way*, *you will envy her and feel that you also want to feel what she felt… She will find a way to put it so nicely such that some of us will just say*: *“Tomorrow*, *I think I will go taste this… I will even skip class…” We just go and experiment so that we can also feel what they have been telling us–*Girl, FGD 5

*If you do not have sex with them (girls)*, *they will call you different names and say ‘usigwadzi’ (‘you can’t propose love’) … they will ask you*: *“Why don’t you do it [sex]*? *‘Unenovi’ or ‘Usidzidzidzi’ (‘you are sexually passive’)*. *Obviously*, *you will end up engaging in sex just to impress your friends—*Boy, FGD 6

*Some of our friends do not accept it when we tell them that we have girlfriends*. *So*, *I will be forced to engage in sex and even take some pictures with my girlfriend while in bed to show them to my friends as proof that I did it… That is the kind of pressure that makes us start having sex early–*Boy, FGD 7

### Theme 4: Contextual factors

Participants stated that some neighborhoods are associated with high levels of delinquent behavior such as substance or drug use, such as in rural areas where marijuana is cultivated, which influences them to start sex early. For example, they stated that living in rural areas puts them at unique risks because they walk long distances to school through bushes, and fetch water from wells, which presents them with opportunities to engage in sexual activities. They also mentioned that changing location from rural to urban; clubbing or attending night parties (especially in urban areas), attending prom nights and soccer games, and staying in a homestead that brews traditional beer put them at risk of early sexual debut:

*There are a lot of drugs in the rural areas…once high*, *they make someone crave sexual pleasure… One may find himself/herself engaging in sexual intercourse anytime*.–Boy, FGD 2

*We sell traditional beer at home and ladies will come to buy the beer and drink it in the yard… Once they are drunk*, *they fall asleep*, *and you find that I am alone at home*. *So*, *I will take the one who has fallen asleep to my house*, *and I will end up having sex with her*.–Boy, FGD 7

We attend parties where we do so many things. You find that one person approaches you… we end up doing things we never planned to do. We use the available chance and it’s a one-day thing, and if you know that you are meeting this person for the first, and probably last, time, you will do ‘quick things’–Boy, FGD 7

During prom, our parents do everything for us and provide even cars for the girls. When she gets there, she feels free and she won’t sleep at home that night–Boy, FGD 1

*Our area of residence has too many bushes and forests… on our way from school*, *in those bushes*, *we are tempted to have sex… it is very easy for us to do it in the bushes*.–Boy, FGD 7

*In towns*, *there are nightclubs*, *bars and people attend every evening*, *yet a lot of things happen there*, *and some people have sex there—*Girl, FGD 3

### Theme 5: Mass media

In all the FGDs, participants stated that advances in technology (i.e., watching television [TV], cellphone ownership, and having access to social media) expose them to explicit content at younger ages, which makes them want to experiment sex early. Participants stated that daily TV shows and movies incite them to start experimenting with sex early as they showed explicit scenes. They stated that at times they even watch these shows with their parents. Participants also stated that owning a cellphone enabled them to access social media, yet that is where their peers send them nude photos, and they receive invites on Facebook from strangers whom they end up meeting and engaging in sexual activities. They also said that parents who own cell phones usually stay glued to their mobile phones and never have time to talk to them:

You find that I am watching with my mom and dad, and people on TV start kissing, my parents may laugh and then I join in the laughter… what does that mean? Watching stuff related to sex with my parents tells me that my mom and dad do not care that I see this… It means they don’t care even if I do it–Boy, FGD 1

We watch too much TV, yet on the TV, there are some channels that show things that are not good for us as young people. We are also tempted to watch them, and then want to do that ‘thing’ (sex) there and there—Boy, FGD 6

*If your boyfriend is busy making you watch pornography*, *you will have all the feelings then end up having sex because it looks attractive on TV–*Girl, FGD 4

*When our parents come back from work*, *they sit on the bed or couch and say they are really tired*, *and immediately browse through their WhatsApp…They do not ask about the kids*, *and they don’t talk to us …*. *The problem is the technology; it has destroyed our youthful stage*–Boy, FGD 2

*Social media contributes a lot*. *Let me say I joined Facebook or WhatsApp*, *and I post my picture that I think is hotter than any other picture I have ever taken… I will get many likes for my picture and my inbox will be flooded with messages from strangers who will tell me that they want me*. *Maybe one man will even ask to meet me… when I meet him*, *he will ask me to go to his place… when we reach there*, *obviously*, *he will need to have sex with me before I leave–*Girl, FGD 5

*You find children doing Grade 5 owning cell phones… When you browse through the phone you won’t find stuff related to schoolwork*, *instead*, *you will find pictures of naked girls*, *including explicit videos… Definitely*, *that kid will start having sex early–*Boy, FGD 1

### Theme 6: Cultural factors

Participants stated that attending cultural ceremonies or engaging in some cultural practices and traditions promoted early sexual debut as it offered the opportunity for them to be away from home or unsupervised. They listed ceremonies such as attending traditional weddings and *lobola* ceremonies (which usually last for a whole weekend) and attending the annual Reed Dance (*Umhlanga*) ceremony, which usually lasts for a whole week. Interestingly, they also listed attendance of cultural events by parents, such as the annual Marula (*Buganu*) ceremony, to be a contributing factor to their early sexual debut as they are left unsupervised at home, and hence they get a chance to visit or invite their partners:

In the different cultural events that we have in the country, such as traditional weddings (Umtsimba), lobola (‘payment of dowry’), etc., you find that there is a lot of hot stuff like beer, traditional brew, etc. Some youth like to attend those events where they get drunk and when you are drunk, anything can happen as you have no total control over your body–Girl, FGD 5

Certainly, if a girl leaves home, she will tell her parents that she is attending the Reed Dance… She will use that as an opportunity to be away from home for one week. She will look for boys instead of attending the Reed Dance… because she needs money, but these men will need something in return, sex–Boy, FGD 6

At times we spend long periods away from home just like during the Reed Dance… there is no adult to monitor us closely… We sometimes do as we please and no one will notice–Girl, FGD 4

The women that attend the Marula Festival leave their children at home on their own… the children go out as there is no one monitoring them–Girl, FGD 4

However, other participants stated that cultural practices are protective against early sexual debut:

I don’t think our culture has an influence because some cultural practices are no longer even practiced, e.g., arranged marriages, since we now have rights, we choose for ourselves.–Girl, FGD 4

Our culture does not encourage early sexual debut because if a woman marries through the Swazi traditional route (‘Kuteka’), during the ceremony, she is required not to cover the top part of her body so that the spectators in the kraal can assess if she has had sex or not. So, one will want to stay a virgin so that on that day she is not ashamed–Girl, FGD 3

Participants also mentioned that modernization came with the erosion of some of the cultural practices that protected youth from engaging in early sexual debut. They mentioned the abandonment of *Liguma* (for girls) and *Lisango* (for boys), which were traditional fora where mothers and fathers separately talked to girls and boys about different topics, including sex, has resultied in youth not having high sexual efficacy. They also lamented the loss of some of the traditional values, e.g., boys and girls sleeping in separate houses (*Lilawu and Intsanga*, respectively), which were situated away from those of their parents. They said these protected boys from seeing their sisters or mothers walking around wearing towels, which arouse their (boys) sexual emotions:

*You now find that a boy has impregnated his sister*. *Why*? *Because we don’t practice our culture anymore*. *In the past*, *there was a separate sleeping house for girls (Intsanga) and a separate sleeping house for boys (Lilawu)*. *We did not sleep in the same house with our parents*. *However*, *these days*, *our parents build big houses where the whole family stays…We see them (our sisters or female relatives) walking ‘half-naked’ in the house and our sexual desires are aroused*–Boy, FGD 1

*In the past*, *our grandparents used to sit down with us and tell us many things about life and growing up… we lack all that these days… my mother comes back from work very tired; parks her car in the garage*, *goes to the bathroom*, *and thereafter concentrates on her WhatsApp–*Boy, FGD 2

In all FGDs, participants stated that girls who wear revealing clothing tempt boys to start proposing love and asking for sex. Girls mentioned that they have had instances where they were approached by boys who said they saw them while wearing the traditional attire (*indlamu*, a short traditional ‘skirt’) and they liked what they saw, and they would compliment them for having nice bodies and start asking for love. Boys stated that seeing girls in miniskirts during the day makes them want to have sex, as at night, they will have a hard time sleeping as they will fantasize about what they saw during the day, which makes them ‘want to taste’:

*During traditional ceremonies*, *we are supposed to dress up in our full Swazi traditional regalia (Imvunulo)… Obviously*, *there will be someone who will come to me and tell me that he saw me while I was dancing in the arena… He will ask me to be his friend from the very beginning*, *and as we become friends*, *one thing will lead to the next–*Girl, FGD 5

*The way young girls dress up these days is also a factor…*. *They wear revealing clothes that arouse men’s sexual feelings such that they end up craving sex; they will end up tempted and say*, *“I want that girl and would love to taste how she is in bed”*–Boy, FGD 6

*The way that she will be dressed will be so attractive to men*. *Then if I find a chance to do it with her*, *I will not let it go… To some of us*, *having sex is like Christmas; it comes once a year*, *therefore*, *if a chance avails itself*, *we grab it*.–Boy, FGD 6

However, some participants stated that there was nothing wrong with wearing mini-skirts or revealing clothing as, culturally, it is a way for girls to show off their bodies and pride themselves on their chastity:

Culture does not encourage early sex because they always say, a girl should take pride in her body. At home, you don’t get the chance to show off your body while wearing indlamu (a traditional mini skirt) … But at the Reed Dance, people get to see your body, and it is not an embarrassment to be proud of your body … So, this cultural practice tries to teach us that nothing is embarrassing with your body as a girl–Girl, FGD 3

No, revealing clothing is not the issue… clothes were designed to be worn by people, but people should watch where they wear them. Even when going to school, she can wear shorts underneath, but if she wears them in the streets, then that is wrong. She can wear those kinds of clothes (e.g., hot pants) and stay in the house, and if she wants to go out, she needs to find something else to wear… That is a sign of self-respect… Otherwise, if boys see girls in miniskirts, they will want to ‘taste’ because of the way the girls expose themselves to them–Boy, FGD 1

## Discussion

The findings revealed that the reasons for in-school youth to initiate sexual activity early are multifaceted and include peer pressure, parental practices, neighborhood, mass media, and socio-cultural factors. For most of the findings, there were not many differences in the reasons cited by girls from those cited by boys, contrary to findings from studies in the mainstream literature on sexual behaviors [37–39]. Surprisingly, some of the reasons that were mentioned by the youth in this study stem from what would normally be expected to be protective social structures, such as family and religious structures. In this study, the youth stated that attending church activities was one of the reasons for early sexual debut because it presents the them with unsupervised time to engage in sexual activities. The youth reported that at times they leave home under pretenses when they know very well that they were going to meet their sexual partners, similar to findings from a Ghanaian study which found that adolescents used church occasions as opportunities to flee from their strict parents and “chill” with their boyfriends [40]. However, in our study, we had expected that religiosity would be perceived as protective against sexual activities, since nearly 90% of the population practices Christianity [28], a religion that considers premarital sex as a sin. This finding suggests that religion alone may not be protective, however, there may be need for other values to be instilled when socializing children which can help young people resist pressures to engage in early sex.

As a basic socialization unit, family and household processes were reported as central in determining the timing of the first sexual encounter among the youth. Participants cited living arrangements e.g., living alone (e.g., in rented flats), coming from a child-headed home, and staying with non-biological parents as the main drivers of early sexual debut. This could in part be explained by lack of supervision particularly for those who stay alone and those in child-headed households, whereas good communication with parents and good parental monitoring are protective against early sexual initiation [19,26,41]. One intriguing finding in this study was the perceived unconstructive role-modeling by parents and older siblings. Participants stated that they experienced pressure from parents and older siblings who keep on asking them if they have started dating. Participants pointed a finger at their parents as the leading perpetrators of early sexual debut because they display sexual affection, such as kissing in front of their children, yet they lack honest conversations on sex and dating with them [42]. The youth also unequivocally blamed their early sexual activities on poor parental monitoring by their parents and lack of sexuality education. Previous research showed that high-quality mother-child relations are protective against early sexual debut [43]. One study from Eswatini found that high school youth reported low sexual-risk communication with their parents and that such communication was even lower with fathers [30]. The finding on negative role-modeling by parents was surprising since parents are expected to be good role models to their children; an important finding calling for adolescent sexual and reproductive health programmers to engage parents on the adverse effects of negative role parenting.

Poverty was mentioned in all the FGDs as one indirect factor that drives youth, especially orphans, to seek external financial support which exposes them to fall prey to transactional sexual activities [44]. This was also reported in a South African study where the poor economic status of the community would result in youth engaging in transactional sex to be able to afford material things such as cell phones and clothes [42]. Further, the youth in this study attributed their early sexual encounters to what they termed ‘nowadays high-protein diet’, which they believed increased their sex drive. In fact, this is common folklore in Eswatini society; that a protein-rich diet makes young people sexually irrepressible. For example, in the past, in Eswatini food culture, children were prohibited from eating high-protein foods (e.g. eggs, cheese, chicken meat) because it was believed that such prohibitions delayed menarche and subsequently delayed sexual debut [45], hence the youth’s comparison of ‘nowadays’ diet’ vs olden days’ food culture. We could not find any literature on the effect of a high-protein diet on the sex drive of humans. However, animal laboratory research has demonstrated that male *Ceratitis capitate* (a type of Mediterranean fruit fly) that was fed a high-protein diet and exposed to females that were fed a no-protein diet, had a greater number of copulations compared with males that were fed a no-protein diet [46].

The findings on peer pressure were not surprising judging from the overwhelming evidence from previous studies [25,47]. Adolescence is a stage of life where the opinions of peers matter most. For example, in Nigeria, youth stated that they were having early sex because they had been told how pleasurable it was and therefore wanted to experience it themselves [9]. The testing of sexual prowess, especially among boys can be attributed to the youth’s notion of feeling mature; hence they seek ways of evaluating their level of maturity through sex. For example, in South Africa, research revealed that young men seek to assert their masculinity by testing their sexual prowess [48]. Closely related to the testing of sexual prowess, in this study, participants stated that they initiate sexual activities to strengthen their relationship bonds or as a sign of commitment to their partners, to boast about their sex life among peers, and to stay relevant in conversations about sex, similar to findings from a Tanzanian study where youth also stated that they initiated sexual activities to gain respect and acceptance from their peers [41].

The finding on threats from sexual partners was rather surprising, especially if one thinks about the gravity and nature of the threats. Participants stated that they find themselves in transactional sexual partners, usually with older adults because they desire to fit in the lifestyles and to afford the latest fashion trends and luxuries enjoyed by their counterparts from affluent households. Oftentimes, in these intergenerational partnerships, they lose the power to negotiate safe sex or to even refuse sex [49]. The dire consequence of such sexual partnerships is the risk of violence as the girls in this study stated that when they want to terminate such relationships, their partners would hear none of it, and instead, they risk being trafficked, or worse they threaten them with death.Participants in this study also stated that owning a cellphone, watching TV shows and movies, social media, and the internet exposed them to explicit materials which arouse their sexual drives. Boys stated that they access pictures of naked people on their phones which causes them to lust after women, thus leading to the desire to experiment with sex. The link between mass media and early sexual debut is well documented [26,40,47].

We also found that youth thought that the neighborhood one stays in (e.g., near bars or clubs, in rural vs urban, in high substance abuse areas) determines the timing of their sexual debut. Another cited reason was the attendance of social gatherings such as parties and prom nights, similar to findings from elsewhere [40,50]. Lastly, participants’ views on cultural factors were mixed. Eswatini is generally a cultural and traditional society where traditions are honored. Participants stated that when they or their parents attend national and community ceremonies, they (youth) get a leeway “to do as they please”, including drinking alcohol and engaging in sex. Interestingly, some participants stated that national ceremonies like the *Umhlanga* ceremony promoted chastity among girls as they get to show off their bodies and get to be proud of their bodies. These conflicting views highlight the need to go back to the drawing board to evaluate which aspects of the Eswatini culture need re-patterning and reinforcement in order to delay the sexual debut of the Eswatini youth.

### Strengths and limitations

To our knowledge, this is the first study to report on the reasons for early sexual debut in Eswatini. Our sample included both boys and girls, of which the views of both genders are reported which will better inform the planning and design of adolescent sexual and reproductive health interventions aimed at delaying sexual debut in the country. Our sample comprised sexually active youth who were in a better position to provide rich information regarding the phenomenon being studied. That said, the study still has some limitations. First, our sample comprised only in-school youth and from four schools of which the transferability of the findings is limited to the studied population and setting, and therefore, the views expressed by the participants are not representative of all youth in Eswatini, but only to the participants encountered in this study. Second, we could not hold an FGD with girls in one school, and therefore the views of girls are underrepresented in this study. Third, during sampling, girls seemed reluctant to participate in this study compared to boys, evidenced by the one group of girls that did not show up on the day of the FGD in one of the schools. It is possible that the reluctance by girls might have been related to the sensitive nature of the subject matter under study, which might have skewed our sample.

Fourth, we did not triangulate the data collection methods (e.g., conducting individual interviews), of which it is possible that some participants may have hidden their true responses. However, the use of pseudonyms and the fact that participants did not know that they were in the FGD because they had had sex enhanced the quality and depth of the information collected. Fifth, in each FGD, as discussions intensified, participants seemed to be responding to the question “*Why do you think youth*, *in general*, *are sexually active*?” rather than “*Why do you think youth engage in sex early*?” which ended up broadening the scope of the responses. We also did not elicit if early marriage was a culture in the study population during the FGDs, of which we implore future studies to do so. Lastly, our sample was not restricted only to youth who had had an early sexual debut, which means that a majority of the participants did not share their first-hand experiences, but rather their perceptions regarding the phenomenon. The main reason is that it would have appeared discriminatory if we had only put aside pseudonyms of only those who had had an early sexual debut (i.e., <15 years), and that would have meant we were going to end up only with two participants in the study.

## Conclusion and implications

This study revealed that early sexual debut seems to be normalized by young people for several reasons. Some of the social structures such as religiosity, culture, and staying with both parents, which we had expected to be protective against early sexual debut, were cited as catalysts in this study. Other widely mentioned reasons for early sexual debut include peer and partner pressure, mass media, area of residence, changing living environment, staying alone, and low sexual self-efficacy, amongst others. These reasons imply that for youth sexual and reproductive health interventions to have a high impact, they need to take into account the hierarchy of the identified themes within the social and environmental sphere of the youth. The study revealed that family structure, as a basic unit of socialization, has been weakened by the development of social media. Parents were cited as absent and were reported to be negatively socializing their children and were said to be too busy with social media for them to do the much-needed sexuality education.

Strategies for parent-child relational development should be introduced and monitored, especially for those families already experiencing certain household difficulties such as poverty, unstable living arrangements, absence of parents due to work demands, etc. We found that in the context of family and culture, what may seem like a public display of affection on the part of parents to positively influence children, may turn out to be the very reason that drives young people to experiment with what they see their parents doing. These findings call for the need for child-parent communication programs that will restore sexuality education within households. Relevant stakeholders working with orphan and vulnerable children and children in child-headed homes should utilize youth peer social networks and mass media to reinforce SRH messages and implement behavior-change programs for youth. The findings highlight that parents play a major role in influencing youth sexual behaviors and should therefore be directly engaged when designing youth programs.

## Supporting information

S1 Text Socio-demographic and screening/recruitment questionnaire. (DOCX)

S2 Text Semi-structured focus group interview guide. (DOCX)

S3 Text COREQ checklist. (DOC)

## References

[1] Doyle A M., Mavedzenge S N., Plummer M L., Ross DA. The sexual behaviour of adolescents in sub-Saharan Africa: patterns and trends from national surveys. Tropical Medicine and International Health. 2012;17(7):796–807. doi: 10.1111/j.1365-3156.2012.03005.x 22594660

[2] Kangaude GD, Skelton A. (De)Criminalizing adolescent sex: A rights-based assessment of age of consent laws in eastern and southern Africa. SAGE Open. 2018;8(4):2158244018806036. doi: 10.1177/2158244018806036

[3] The Sexual Offences and Domestic Violence Act, No. 15 of 2018, (2018).

[4] Graham P. Against the stream: lowering the age of sexual consent. BJPsych Bull. 2018;42(4):162–4. doi: 10.1192/bjb.2017.26 30025549 PMC6436061

[5] Johnson KA, Tyler K.A. Adolescent sexual onset: an intergenerational analysis. Journal of Youth and Adolescence. 2007;36:939–49. doi: 10.1007/s10964-006-9165-z

[6] Zuma K, Setswe G, Ketye T, Mzolo T, Rehle T, Mbelle N. Age at sexual debut: a determinant of multiple partnership among South African youth. African Journal of Reproductive Health. 2010;14(2):47–54. 21243918

[7] Richter L, Mabaso M, Ramjith J, Norris SA. Early sexual debut: Voluntary or coerced? Evidence from longitudinal data in South Africa—the Birth to Twenty Plus study. South African Medical Journal 2015;105(4):304–7. doi: 10.7196/SAMJ.8925 26294851

[8] Peltzer K, Pengpid S. Early sexual debut and associated factors among in-school adolescents in six Caribbean countries. West Indian Med J. 2015;64(4):351–6. doi: 10.7727/wimj.2014.025 . 26624586 PMC4909066

[9] Omolola FF, Olusegun FA, A OO, Ebun AS, Olufemi AT, Paulin OI, et al. Prevalence and predictors of early sexual debut among adolescents in Ogbomoso, Nigeria. American Journal of Public Health Research. 2018;6(3):148–54. doi: 10.12691/ajphr-6-3-4

[10] Chiao C, Ksobiech K. The influence of early sexual debut and pubertal timing on psychological distress among Taiwanese adolescents. Psychology, Health & Medicine. 2015;20(8):972–8. doi: 10.1080/13548506.2014.987147 25495948

[11] Berry L, Hall K,. Age at sexual debut. In: Institute C, editor. University of Cape Town: Childrens Institute; 2007–2011.

[12] Government of the Kingdom of Eswatini. Swaziland HIV Incidence Measurement Survey 2 (2016–2017). Final Report. Mbabane, Eswatini: 2019.

[13] Central Statistical Office (CSO). Swaziland Multiple Indicator Cluster Survey 2014 Final Report. Mbabane, Swaziland: Government of the Kingdom of Swaziland, 2016.

[14] Central Statisctics Office (CSO) and Macro International Inc. Swaziland Demographic and Health Survey 2006–07. Mbabane: 2008.

[15] NERCHA. Swaziland HIV Prevention Policy. Mbabane: NERCHA; 2012.

[16] Stern E, Cooper D. Experiences and conceptualizations of sexual debut from the narratives of South African men and women in the context of HIV/AIDS. African Journal of AIDS Research. 2014;13(2):121–31. doi: 10.2989/16085906.2014.943252 25174629

[17] UNFPA. UNFPA Annual Report 2014. New York: United Nations, 2015.

[18] Kangmennaang J, Mkandawire P, Luginaah I. Determinants of risky sexual behaviours among adolescents in Central African Republic, Eswatini and Ghana: evidence from multi-indicator cluster surveys. African Journal of AIDS Research. 2019;18(1):38–50. doi: 10.2989/16085906.2018.1552600 30880582

[19] Kastbom AA, Sydsjö G, Bladh M, Priebe G, Svedin C-G. Sexual debut before the age of 14 leads to poorer psychosocial health and risky behaviour in later life. Acta Paediatrica. 2015;104(1):91–100. doi: 10.1111/apa.12803 25213099 PMC4480657

[20] Stryhn JG, Graugaard C. The age at first intercourse has been stable since the 1960s, and early coital debut is linked to sexual risk situations. Ugeskrift for laeger. 2014;176(37):V01140063. 25294037

[21] Shrestha R, Karki P, Copenhaver M. Early Sexual Debut: A Risk Factor for STIs/HIV Acquisition Among a Nationally Representative Sample of Adults in Nepal. Journal of community health. 2016;41(1):70–7. doi: 10.1007/s10900-015-0065-6 26184108 PMC4715759

[22] Baumgartner JN, Geary CW, Tucker H, Wedderburn M. The influence of early sexual debut and sexual violence on adolescent pregnancy: a matched case-control study in Jamaica. International Perspectives on Sexual and Reproductive Health. 2009;35(1):21–8. doi: 10.1363/ifpp.35.021.09 19465345

[23] de Looze M, van den Eijnden R, Verdurmen J, Vermeulen-Smit E, Schulten I, Vollebergh W, et al. Parenting practices and adolescent risk behavior: rules on smoking and drinking also predict cannabis use and early sexual debut. Prevention science: the official journal of the Society for Prevention Research. 2012;13(6):594–604. doi: 10.1007/s11121-012-0286-1 22960939 PMC3505510

[24] de Looze M, Harakeh Z, van Dorsselaer SA, Raaijmakers QA, Vollebergh WA, ter Bogt TF. Explaining educational differences in adolescent substance use and early sexual debut: the role of parents and peers. Journal of adolescence. 2012;35(4):1035–44. doi: 10.1016/j.adolescence.2012.02.009 22418451

[25] Durowade KA, Babatunde OA, Omokanye LO, Elegbede OE, Ayodele LM, Adewoye KR, et al. Early sexual debut: prevalence and risk factors among secondary school students in Ido-ekiti, Ekiti state, South-West Nigeria. Afr Health Sci. 2017;17(3):614–22. doi: 10.4314/ahs.v17i3.3 29085388 PMC5656187

[26] Kassahun EA, Gelagay AA, Muche AA, Dessie AA, Kassie BA. Factors associated with early sexual initiation among preparatory and high school youths in Woldia town, northeast Ethiopia: a cross-sectional study. BMC Public Health. 2019;19:378. doi: 10.1186/s12889-019-6682-8 30947690 PMC6450012

[27] Polit DF, Beck CT. Nursing research: Generating and assessing evidence for nursing practice. 10th ed. Philadelphia: Lippincott Williams & Wilkins; 2017.

[28] Central Statistical Office (CSO). The 2017 population and housing census vol 3. Mbabane, Eswatini: Government of the Kingdom of Eswatini, UNFPA; 2019.

[29] Swaziland Human Development Report. HIV and AIDS and culture. 2007.

[30] Shongwe MC, Chung M-H, Chien L-Y, Chang P-C. Does parenting style moderate the relationship between parent-youth sexual risk communication and premarital sexual debut among in-school youth in Eswatini? PLoS ONE. 2021;16(1):e0245590. doi: 10.1371/journal.pone.0245590 33493222 PMC7833135

[31] Lincoln Y, Guba E. Naturalistic Inquiry. Newbury Park, London, New Delhi: Sage Publications Inc.,; 1985.

[32] Guba EG, Lincoln YS. Competing paradigms in qualitative research. Handbook of qualitative research. Thousand Oaks, CA, US: Sage Publications, Inc; 1994. p. 105–17.

[33] Shenton AK. Strategies for ensuring trustworthiness in qualitative research projects. Education for Information. 2004;22(2):63–75. doi: 10.3233/EFI-2004-22201

[34] Tong A, Sainsbury P, Craig J. Consolidated criteria for reporting qualitative research (COREQ): a 32-item checklist for interviews and focus groups. Int J Qual Health Care. 2007;19(6):349–57. doi: 10.1093/intqhc/mzm042 17872937

[35] IBM Corp. IBM SPSS Statistics for Windows, Version 20.0. Armonk, NY: BM Corp.; 2011.

[36] SocioCultural Research Consultants LLC. Dedoose, web application for managing, analyzing, and presenting qualitative and mixed method research data. 8.2.14 ed. Los Angeles, CA: SocioCultural Research Consultants, LLC; 2019.

[37] Pringle J, Mills KL, McAteer J, Jepson R, Hogg E, Anand N, et al. The physiology of adolescent sexual behaviour: A systematic review. Cogent Social Sciences. 2017;3(1):1368858. doi: 10.1080/23311886.2017.1368858 29201945 PMC5692360

[38] Kempadoo K, Dunn LL. Factors that shape the initiation of early sexual activity among adolescent boys and girls: A study in three communities in Jamaica. Kingston: UNICEF and UNFPA. 2001.

[39] Cubbins LA, Tanfer K. The influence of gender on sex: A study of men’s and women’s self-reported high-risk sex behavior. Archives of Sexual Behavior. 2000;29(3):229–57. doi: 10.1023/a:1001963413640 10992980

[40] Adongo BW. Assessing factors influencing early sexual initiation among adolescents (13 to 19 years) in Ghana: a qualitative study. International Journal of Caring Sciences. 2018;11(1):53–60.

[41] Mmbaga EJ, Leonard F, Leyna GH. Incidence and predictors of adolescent’s early sexual debut after three Decades of HIV interventions in Tanzania: A time to debut analysis. PLoS ONE. 2012;7(7):e41700. doi: 10.1371/journal.pone.0041700 22848571 PMC3407234

[42] Khuzwayo N, Taylor M. Exploring the socio-ecological levels for prevention of sexual risk behaviours of the youth in uMgungundlovu District Municipality, KwaZulu-Natal. African Journal of Primary Health Care & Family Medicine. 2018;10(1):1–8. doi: 10.4102/phcfm.v10i1.1590 29781679 PMC5843945

[43] Nogueira Avelar e Silva R, van de Bongardt D, van de Looij-Jansen P, Wijtzes A, Raat H. Mother–and father–adolescent relationships and early sexual intercourse. Pediatrics. 2016:e20160782. doi: 10.1542/peds.2016-0782 27940677

[44] Chae S. Timing of orphanhood, early sexual debut, and early marriage in four sub-Saharan African countries. Studies in Family Planning. 2013;44(2):123–46. doi: 10.1111/j.1728-4465.2013.00349.x 23719999 PMC4000696

[45] Nsibande A. Grade 1 pupil among 4 570 teenage pregnancy dropouts: ’Chicken meat, eggs make girls fertile at early age’. Times of Eswatini. 2019 28 January 2020;Sect. News.

[46] Joachim-Bravo IS, Anjos CS, Costa AM. The role of protein in the sexual behaviour of males of Ceratitis capitata (Diptera: Tephritidae): mating success, copula duration and number of copulations. Zoologia (Curitiba). 2009;26:407–12.

[47] Lee RLT, Yuen Loke A, Hung TTM, Sobel H. A systematic review on identifying risk factors associated with early sexual debut and coerced sex among adolescents and young people in communities. Journal of Clinical Nursing. 2018;27(3–4):478–501. doi: 10.1111/jocn.13933 28639335

[48] Harrison A, Cleland J, Gouws E, Frohlich J. Early sexual debut among young men in rural South Africa: heightened vulnerability to sexual risk? Sexually Transmitted Infections. 2005;81(3):259. doi: 10.1136/sti.2004.011486 15923298 PMC1744981

[49] Nkosana J, Rosenthal D. The dynamics of intergenerational sexual relationships: the experience of schoolgirls in Botswana. Sexual Health. 2007;4(3):181–7. doi: 10.1071/sh06070 17931531

[50] Ahimbisibwe J. Predictors of early sexual debut among adolescents in North Norway: The Arctic University of Norway; 2014.

