# Supplementary material for: “Our parents kiss in front of us”: Reasons for early sexual debut among in-school youth in the Manzini Region in Eswatini
Source: PLoS One. 2023 Mar 10;18(3):e0282828. doi: 10.1371/journal.pone.0282828 (PMC10004614; doi:10.1371/journal.pone.0282828)
Supplement: S1 Text — (DOCX) [file pone.0282828.s001.docx]

Appendix 3A

**Demographic questionnaire**

**Interviewer’s Name ……………………………….**

**Study ID ……………………………………………**

**Date of interview …………/……………/…………**

**(dd/mm/yyyy)**

**Tick the selected response**

1. Age [ ] years
2. What is your gender?

Male [ ]

Female [ ]

1. What part of Manzini do you live?

Urban [ ]

Rural [ ]

1. What class are you in?

Form 1 [ ]

Form 2 [ ]

Form 3 [ ]

Form 4 [ ]

Form 5 [ ]

1. What is your religion?

Christian [ ]

Other (specify) [ ]

……………………………………………………..

1. Who are you currently staying with?

Both parents [ ]

Father only [ ]

Mother only [ ]

Child headed [ ]

Guardian [ ]

Other [ ]

Specify……………………………………….

1. How many people are in your household?

< 3 people [ ]

4-6 people [ ]

More than 7 people [ ]

1. What is your average monthly pocket money from home?

E0 [ ]

E1- E20 [ ]

E21-E49 [ ]

E 50 and above [ ]

1. Have you ever had sexual intercourse?

Yes [ ]

No [ ]

If No to number 9, End interview here

1. If yes to number 9, was it (tick all that are applicable)

Vaginal [ ]

Anal [ ]

Oral [ ]

1. If yes to number 9, at what age did you first have sex? [ ]
2. If yes to number 9, was your first sexual intercourse ( select the one that is applicable)

Voluntary [ ]

Forced [ ]

Thank you.
