# Supplementary material for: “Our parents kiss in front of us”: Reasons for early sexual debut among in-school youth in the Manzini Region in Eswatini
Source: PLoS One. 2023 Mar 10;18(3):e0282828. doi: 10.1371/journal.pone.0282828 (PMC10004614; doi:10.1371/journal.pone.0282828)
Supplement: S2 Text — (DOCX) [file pone.0282828.s002.docx]

**Appendix 4A**

**Focus Group Interview Guide**

**Thank participants for being participants in the FGD and assure them that what will be discussed will remain confidential as stipulated in the IC form.**

Ask this main question: What do you think are the reasons that youth have for engaging in early sexual intercourse?

**Probing questions**

***The following questions will help you probe further on what the learners have already mentioned as reasons***

What is your feeling about….?

Can you tell me more about…?

What about…..?

Why do young people……?

And more…

**Thank you for sharing with us. We hope this will help in improving programmes for youth.**
